# Capturing mixture composition: an open machine-readable format for representing mixed substances

**DOI:** 10.1186/s13321-019-0357-4

**Published:** 2019-05-23

**Authors:** Alex M. Clark, Leah R. McEwen, Peter Gedeck, Barry A. Bunin

**Affiliations:** 1 Collaborative Drug Discovery, Burlingame, CA USA; 2 Cornell University, Ithaca, NY USA

**Keywords:** Mixture, Mixfile, Molfile, InChI, MInChI

## Abstract

We describe a file format that is designed to represent mixtures of compounds in a way that is fully machine readable. This *Mixfile* format is intended to fill the same role for substances that are composed of multiple components as the venerable *Molfile* does for specifying individual structures. This much needed datastructure is intended to replace current practices for communicating information about mixtures, which usually relies on human-readable text descriptions, drawing several species within a single molecular diagram, or mutually incompatible ad hoc solutions. We describe an open source software application for editing mixture files, which can also be used as web-ready tools for manipulating the file format. We also present a corpus of mixture examples, which we have extracted from collections of text-based descriptions. Furthermore, we present an early look at the proposed IUPAC Mixtures InChI specification, instances of which can be automatically generated using the *Mixfile* format as a precursor.

## Introduction

In the world of practical chemistry it is rare to encounter a substance that could be unconditionally described as absolutely pure, i.e. exclusively one molecular structure. Most samples of a singular compound that are purchased from commercial vendor have an estimated purity, and many reagents are solutions, adducts or mixtures of isomers. Outside of the laboratory there are frequent encounters with substances that feature distinct chemical entities, such as pharmaceutical drug formulations, cleaning products such as shampoos, hydrocarbon cocktails for automotive transport, etc.

Since the 1980s, the use of computer software to represent organic molecules has been routine within the drug discovery industry, with a number of machine readable formats gaining considerable traction (e.g. Molfile [1], SMILES [2] and InChI [3]). This has been a cornerstone of a vibrant cottage industry that is used to manage structure–activity information about small molecule drug candidates, and brings together a large number of modelling, simulation and prediction techniques. In spite of the demonstrable success of these tools, file formats and identifiers for describing distinct molecule species (and to a lesser extent chemical reactions based on them), there remains to this day no widely used protocol for representing these molecules in the form that they are encountered in the real world, i.e. mixtures.

Communication of information about mixtures is usually done using plain text descriptions, which are usually meaningful to an expert. Many of the mixture annotations are quite simple, e.g. a purity indicator as a percentage, or the active ingredient followed by its concentration within a solvent or mixture thereof. There are enough common patterns in use that one could imagine a small set of rules that could extract the key components for most mixture descriptions, but as with all cases where the full domain of natural language is allowed, there will always be residual edge cases that defy correct classification by even the most sophisticated algorithm.

One partial solution that has been adopted by certain custom databases, especially those for managing inventories, is to use a single-entity format for capturing the compounds of interest: the active entity is described using Molfile, SMILES, InChI and/or other related descriptions, and the material as a whole is represented as text. Sometimes other metadata can be captured (e.g. purity, or solvent) which can provide an adequate description for many simple mixtures, which for many collections is the majority. However, even when a handful of fields is sufficient for the task at hand, this does not address the issue of interoperability. Because there is no industry standard protocol for representing mixtures, any two custom databases are unlikely to be able to share their content in a meaningful way—not at least without a bespoke import process, with a high likelihood of having incompatible definitions.

Another workaround that is encountered frequently is the inclusion of multiple structures within a single representation (e.g. *xylenes* drawn as a 3-component structure with each of the *ortho*, *meta* and *para* isomers). This is problematic because there is usually no indication that these are independent entities, and that they are not stoichiometric. On the other hand, it is sensible to represent adducts in a single structure, especially when the constituents are chemically associated with each other (Na^+^Cl^−^ being an obvious example, with compounds that have solvent-of-crystallization at the time the mixture was formulated being applicable also). For practical purposes, it is generally useful to think of each component of the mixture as a distinct stoichiometric molecular entity that could, in principle at least, be isolated in its pure form.

There is a clear need for an industry standard format that can capture a broad range of chemical materials that are present in laboratories, and ideally other scenarios as well. Such a format needs to be simple and concise, and able to effectively describe a large number of substances without overloading too much complexity. One working definition is that a mixture format should be able to capture the known ingredients of a material, as they were at the time of mixing, and for this purpose there are three essential properties:

- compound
- quantity
- hierarchy

The *compound* should be represented by a chemical structure whenever it is appropriate, with a name provided also when possible (e.g. *benzene* can be represented by both its common name and a sketch of the iconic hexagonal structure, whereas *gelatinous coal tar* cannot be resolved to a molecular entity, so can be described by name only).

The *quantity* can be represented in several ways, but is typically a concentration of some sort (e.g. percentage, ratio, molarity, etc.). Mixtures often contain uncertainty in various forms, e.g. concentration can be provided as a range, or a minimum value. Often a quantity will be unknown, or it will be implied as the remainder of the content, which is common for solvents. Ratios add up to less than 100% in cases where a purity is provided, and the actual nature of the impurities is not known: these unknowns are presumed to make up the difference.

The *hierarchy* of a mixture is necessary for capturing materials of intermediate complexity. A common example of where this is necessary is when an active ingredient is dissolved in a solvent mixture. For example, consider the description *1*-*methylpiperidine 15% in methanol/water (1:2)*: the nature of the mixture is best captured as:

- 1-Methylpiperidine (15%)
- *solvent*:
  - water (1/3)
  - methanol (2/3)

The basic mixture requirements (compound, quantity and hierarchy) can be used to capture the composition of most of the chemical stockroom inventories and vendor catalogs, in addition to many consumer goods and pharmaceutical formulations. In addition to these basic properties, the datastructure can be augmented to include any number of additional metadata fields that are useful. For example: synonyms and extended text descriptions; database identifiers; links to resources; physical properties; handling codes and expiry dates, etc.

The main reason for even considering updating an information system to be able to describe mixtures in this more formal way is that the inherent machine readability opens the door to informatics tasks that were not previously possible. For example, once the structures that make up a mixture are all represented, it becomes possible to perform structure searches for any of the components, or to group them by their given substituents. It is also possible to cross reference these substances to hazard databases, so that safety information can be automatically obtained from an authoritative source, rather than re-entered as a line item for each stockroom instance [4].

Standardisation of mixture definitions is incredibly valuable, especially since the most common methodology—plain text—so frequently results in similar materials being described with different keywords that are intractable to software analysis. Recasting mixtures into a format that is a collective superset of the molecular definitions of its components means that all of the technologies that have been developed within the cheminformatics industry are applicable to the components individually as well as collectively. One in particular has high applicability: the InChI identifier can be calculated from any described structure, which can be leveraged for many and diverse purposes (e.g. most databases allow lookup via InChI, and many cheminformatics tasks can be performed with trivial string manipulation functions).

In this article we introduce the *Mixfile* format, which is intended to be to mixtures what the *Molfile* format is to molecules. We describe its composition and use cases, an open source editor and tools, its use to generate Mixtures InChI (*MInChI*) strings, and preliminary extraction from plain text to create a baseline dataset.

## Results and discussion

### Definition

**Fig. 1:**
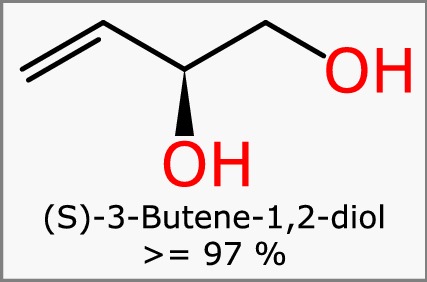
A simple mixture with a single known component, (S)-3-butene-1,2-diol, which has a purity estimate

The simplest kind of Mixfile represents a mixture that is essentially a single component with a purity value, as shown in Fig. 1. The singular component is described by three pieces of information: the structure of the butene derivative, its name, and the concentration which is given as ≥ 97%. This representation only requires a single component because the impurities are unknown, and thus unspecified. This simple example represents a use case that is incredibly common, especially within reagent catalogs.

**Fig. 2:**
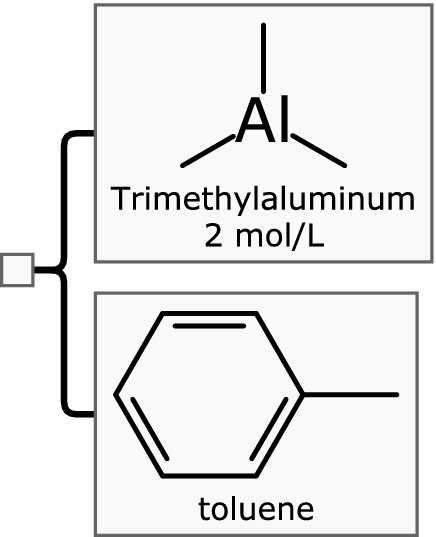
A two component mixture with an active ingredient (trimethylaluminium) with known concentration dissolved in a solvent (toluene)

Another very common use case is when the active ingredient is provided as a solution, as shown in Fig. 2. In this case, the hierarchical nature of the Mixfile format is invoked. The root node is blank, although it can be used to store secondary metadata about the mixture overall. It contains two components: the active ingredient and the solvent. Both of them are represented by name and structure. The active ingredient, *triethylaluminium*, is indicated to be 2 molar. The concentration of the solvent, *toluene*, is left blank, which by convention means that it makes up the remainder of the mixture. While it would be valid to calculate the molarity of the solvent and include this information, it is superfluous, and for convenience and representational clarity, is better left out.

**Fig. 3:**
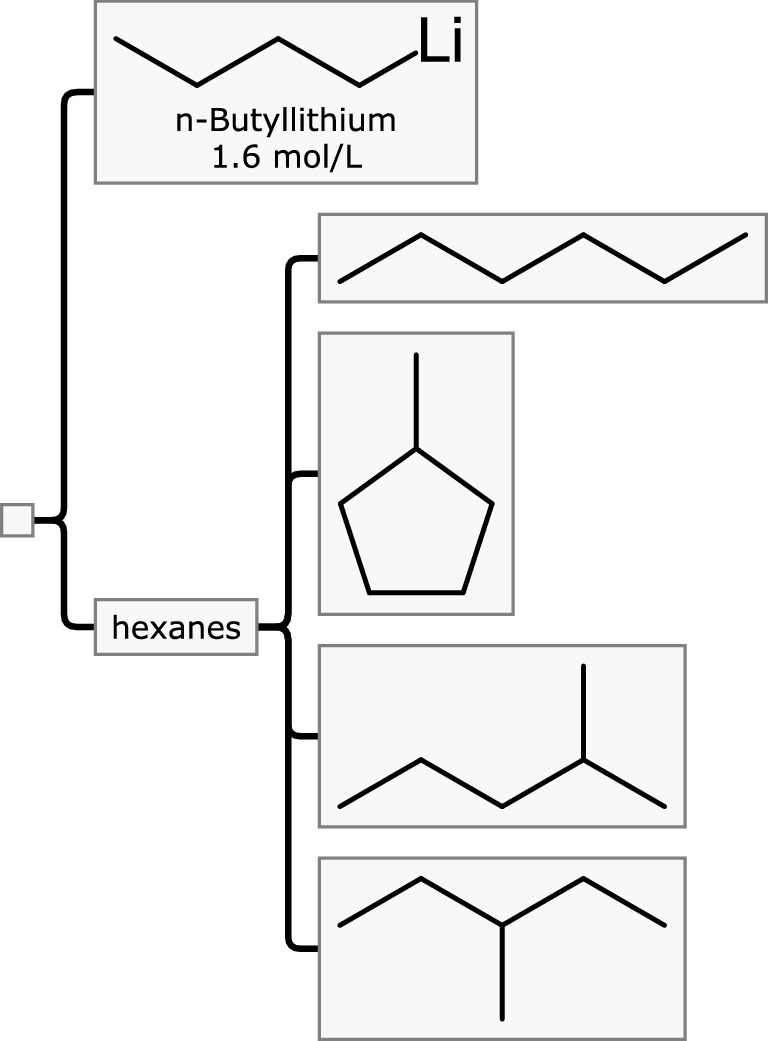
Butyllithium dissolved in “hexanes”, which is itself a mixture, made up of known compounds of unspecified proportions

Mixfile hierarchies have no limit to their depth or height, and use of nesting is a convenient way to express mixtures-of-mixtures. For example, consider *n*-*butyl lithium* dissolved in the solvent that is colloquially referred to as *hexanes*, shown in Fig. 3.

This particular choice of hierarchical description clearly indicates that the substance being described is a mixture of two distinct *things*: the reagent and the solvent. The solvent occupies one container node, which is described using the name *hexanes*. Because it is itself a mixture, it does not have a structure, and it is also not given a concentration (since it is implied to constitute everything other than the reagent). The *hexanes* component has four sub-components assigned to it, which represent the major C_6_ isomers that make up the solvent. If the relative proportions of the isomers were known, they could be expressed as concentrations (e.g. as a ratio, or volume/mass/molar percentages), but in this case, the proportion is not provided by the manufacturer. As such it illustrates that the Mixfile format is comfortable with incomplete data, which is important since it would be incorrect to insist on providing information which is not available.

**Fig. 4:**
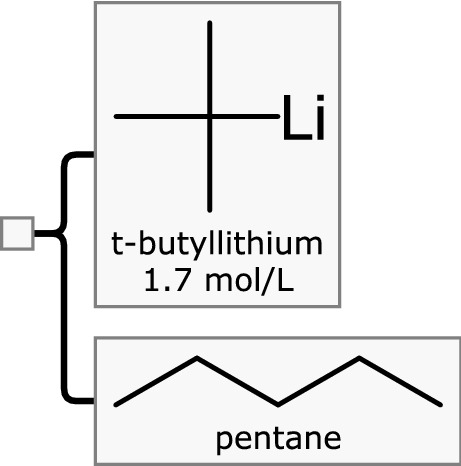
Tert-butyllithium in pentane, for which the choice of solvent is especially important for safety purposes

One very practical reason for taking the effort to describe substances such as organolithium reagents is that the safety and hazards vary based on composition. Consider the related and much more dangerous *tertiary* butyl lithium reagent, which is shown in Fig. 4.

Knowledge of the active ingredient alone (*t*-butyllithium) is sufficient to ascertain that this material is pyrophoric, since it has this characteristic in all of its forms. For *n*-butyllithium, however, solutions are pyrophoric only at higher concentrations (ca. 10 mol/L and above) [5]. Therefore being able to keep track of the active ingredient *and* its concentration is essential for being able to provide appropriate safety, handling and disposal advice. In the case of these two organolithium reagents, the solvent composition is also important, e.g. *t*-butyllithium is commonly sold as either pentane or heptane solutions, and these solvents have drastically different volatility, which is a very important detail for a mixture that bursts into flame on contact with air. Any hazard database would be incomplete (and possibly dangerous by omission) without the ability to store and match all of these facts.

Another important consideration with highly reactive reagents like organolithium solutions is that they decay over time and need to be titrated [6, 7] to redetermine the concentration. This means that it is not sufficient to mark samples with a reference to the properties that it had at time of purchase, rather it needs to be recorded with a datastructure that can capture the changing concentration, and ideally do so in a way that can be useful (e.g. combine with reaction planning software to calculate the volume required for stoichiometric use).

**Fig. 5:**
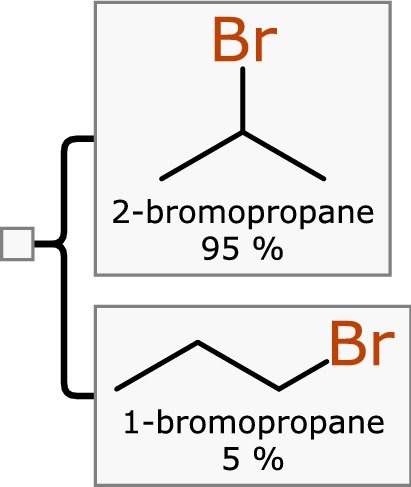
Two isomers resulting from bromination of propene, represented as a mixture with their relative proportions indicated

The component hierarchy can also be used to represent mixtures of isomers, which is a common use case for the outcomes of reactions that are not followed by an effective purification step, e.g. the result of Markovnikov addition [8] of bromine, shown in Fig. 5.

While some kinds of isomers can be effectively represented within the structure of a single component (such as racemic stereoisomers), enumeration is often preferable even when there are alternatives. Enumeration has some advantages over more concise encoding options, e.g. the visualisation is very clear, assignment of relative concentrations is straightforward, and the implementation is simple.

**Fig. 6:**
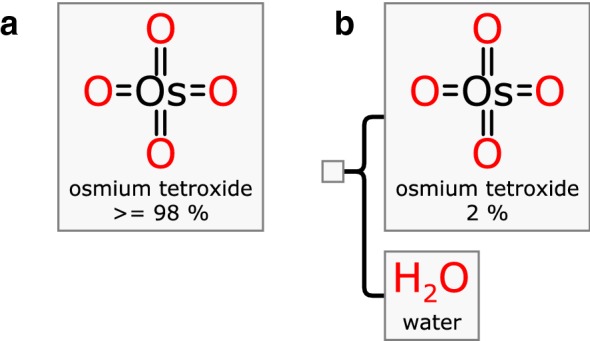
Osmium tetroxide in two forms: **a** neat and **b** solution, which have very different safety profiles

Recording information about the properties of mixtures is important for a great many reasons, not least of which is safety. For example, consider two commercially available forms of osmium tetroxide, shown in Fig. 6. The Mixfile represented in (a) is the solid form which is mostly pure, while (b) is the same active ingredient as a dilute solution in water. Both of these materials are extremely toxic, but the instructions for storing, handling and disposing of them are quite different. Without a well defined machine readable format for drawing the distinction between the raw solid and the dilute solution, locating the right material safety datasheet would be dependent on the knowledge and experience of the scientist performing the lookup. Another poignant example is sodium azide, which is extremely toxic in its pure solid form [9] but when dissolved in water at concentrations of lower than 0.1% it is considered benign enough to use as a food preservative [10].

**Fig. 7:**
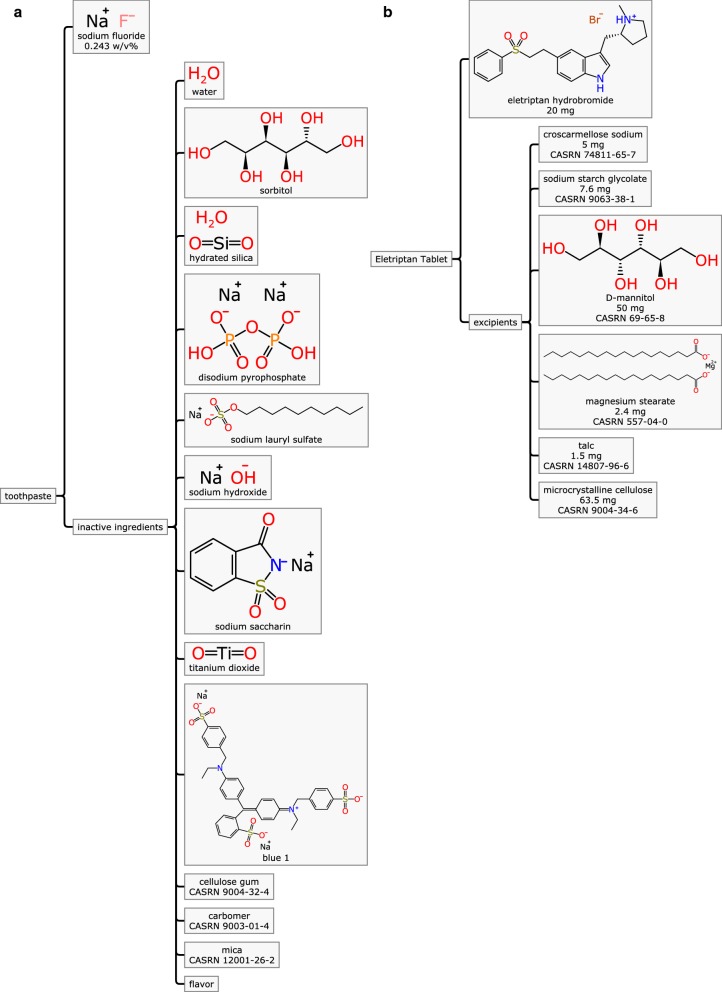
Two mixtures that are common household items: **a** a brand of toothpaste, and **b** a formulation of eletriptan

Mixture descriptions are also relevant outside of the chemical laboratory, since there are innumerable consumer products that could benefit from descriptions with detailed metadata, such as is shown in Fig. 7. Example (a) describes a common brand of toothpaste, while (b) is a tablet formulation for *eletriptan* [11]. Both of these household products have common characteristics in terms of how the mixtures are defined: each of them has an active ingredient (*sodium fluoride* and *eletriptan hydrobromide* respectively) and a host of inactive ingredients. The active ingredients are usually the focus of these consumer products, but the additional materials that are added are very important: they typically impart characteristics that affect stability, texture, flavour and efficacy. They are also common sources of concern regarding toxicity and unwanted side effects, and so compiling accurate, complete and machine readable data for all of the constituents is important, not least of all because it would be possible to quickly identify all such consumer products with any particular component in question whenever there are health concerns. From the R&D perspective, drug formulation is an empirical process: the exact composition and amount of each excipient is an essential characteristic of a drug tablet, and so accurately recording all experimentally determined formulations and pairing them with their effective efficacy is an essential part of product design.

For consumer products even more often than laboratory reagents, some portion of the constituents may not be readily represented by one-or-several distinct chemical structures. Recognition of this limitation is a key design consideration of the Mixfile: in these cases, whatever metadata is available should be provided. There is typically an available name of some form, and sometimes references to external databases that contain information about mixtures, e.g. the Chemical Abstracts Registry Number (CASRN) [12] is often used. These references are not inherently machine readable, and so must be thought of as a placeholder: facilitating a non-automated fallback is preferable to omitting the information entirely, and part of the future work for this project is to expand the ability to describe more complicated structure fragments, like polymers.

### Software

**Fig. 8:**
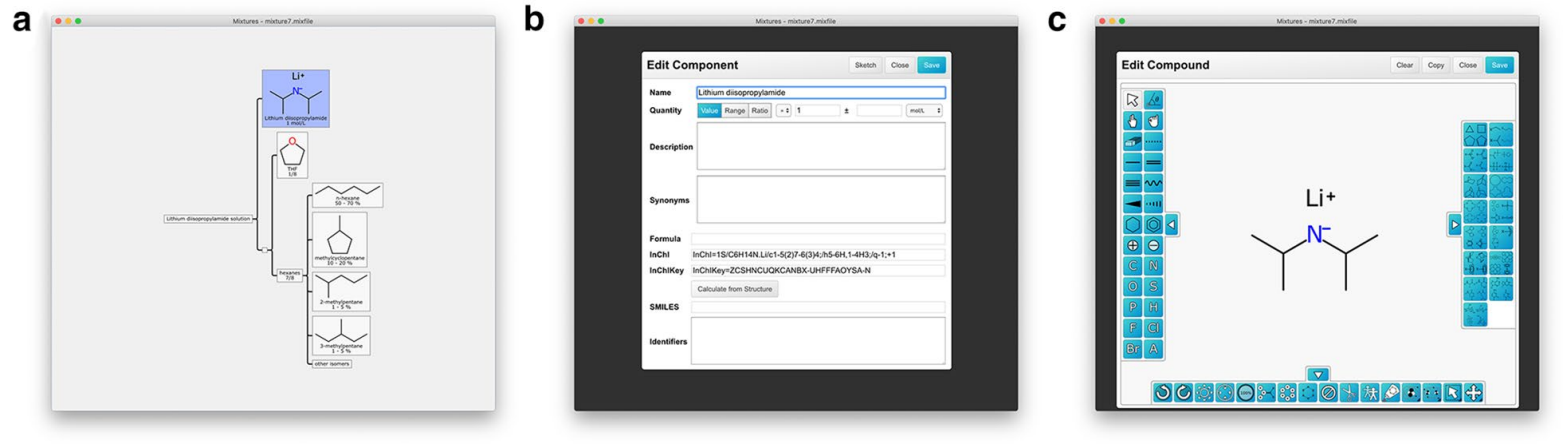
Screenshots of the mixture editor: **a** mixture overview, **b** component editor, **c** structure sketcher

In order to make use of the Mixfile format, we have created a straightforward editor that can be used to define mixtures. Figure 8 shows several panels: the main editor window (a), represents the hierarchical outline of the mixture. The components that make up this tree can be added, deleted, moved, edited, etc., using conventional menu, mouse and keyboard shortcuts. Editing individual components brings up either of two dialogs: one for general details (b) and another for sketching the structure (c).

The mixture editor has the ability to invoke the calculation of InChI strings for any of the constituent structures, which is done via the standard command line tool (which is installed separately [13]). As described subsequently, it also has the ability to create the correspondingly derived MInChI notation for the mixture.

**Fig. 9:**
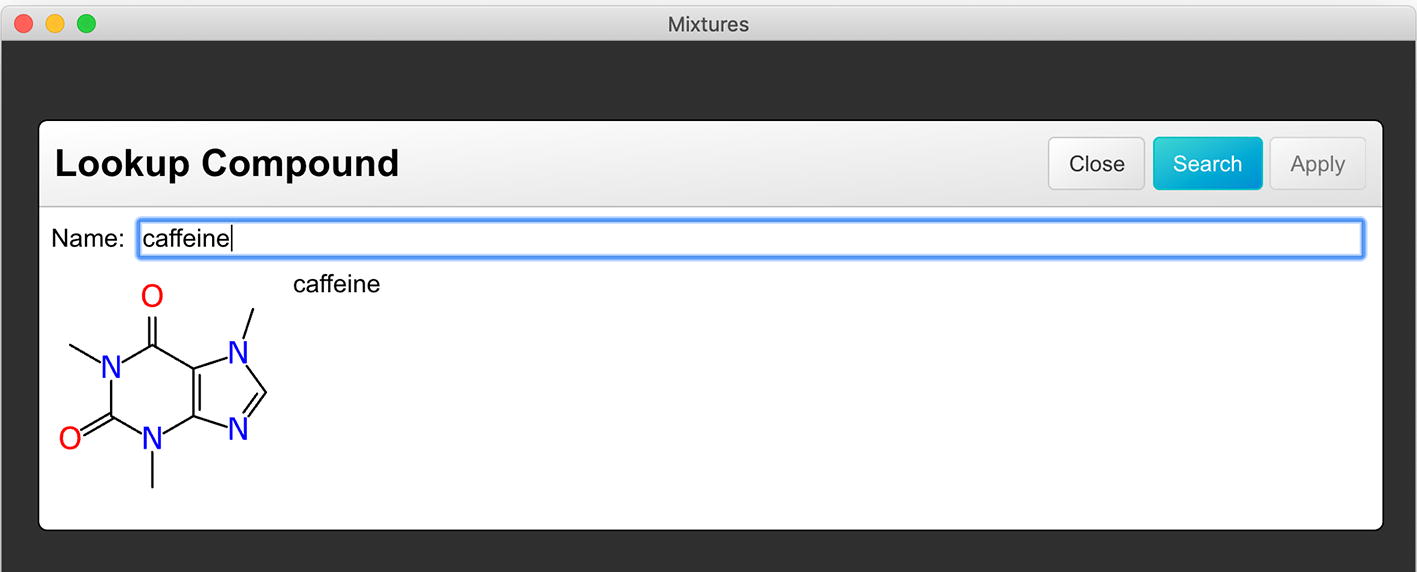
Screenshot of the database lookup feature

As the Mixfile project evolves, the editor will be improved incrementally, and the latest developments will continue to be made available as open source software. One example of an additional utility feature is the ability to lookup structures by name in an external database, shown in Fig. 9. This is a convenient way to fetch structures for which the name is known, so as to avoid having to draw or locate-and-paste the corresponding sketch. At the time of submission, only PubChem is supported, though this could easily be extended to support other databases.

While the best case scenario for generation of machine-readable metadata is to have it created directly by the originating scientist in a format that can express all of the details, the fact is that almost all of the existing mixture information is expressed as text. These text descriptions are usually quite understandable to humans, although on occasion the chosen syntax can be ambiguous, even to an expert. Many of these text descriptions occur within long form paragraphs (e.g. literature publications), but they are quite often abstracted out with a clearly defined beginning and ending: this is observed frequently in online vendor catalogs (e.g. Sigma-Aldrich [14] ThermoFisher [15] Alfa Aesar [16] and many others) and within bespoke chemical inventory systems.

**Fig. 10:**
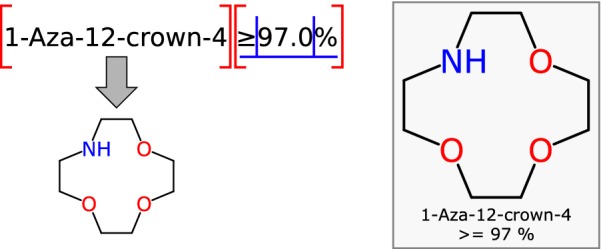
A parsing step for text-to-mixture analysis being applied to a single chemical name with an accompanying purity estimate

It is possible to compose a set of rules that can interpret a large proportion of mixtures from such a dataset. Consider a simple example such as “1-Aza-12-crown-4 ≥ 97.0%”, which describes a single known compound that makes up the majority of the material, and by implication, some number of unknowns that make up the remainder. A parsing operation can be graphically depicted, as shown in Fig. 10. The first rule ascertains that *1*-*Aza*-*12*-*crown*-*4* is the name of a chemical entity which can be mapped to a structure definition. The second rule determines that ≥ 97.0% is a quantity definition which provides *relation*, *value* and *units*.

**Fig. 11:**
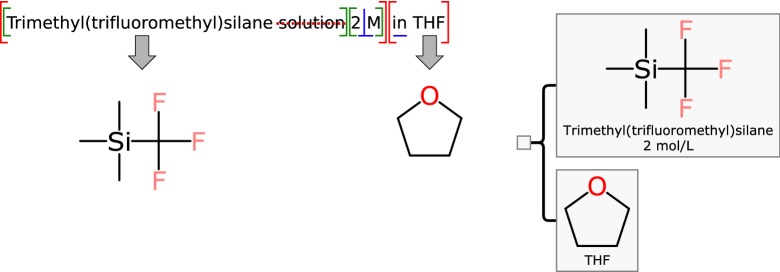
Text parsing rules being applied to a mixture that is partitioned separately into active ingredient and solvent

Mixfiles for which multiple components are defined explicitly require more parsing steps. The most common laboratory examples being reagent-in-solvent pairs, expressed using text such as “Trimethyl(trifluoromethyl)silane solution 2 M in THF”, shown graphically in Fig. 11. In this case the parsing rules need to find the boundary point between the two components, and recursively analyze those. An overall rule of {solute definition} **in** {solvent definition} applies to this example, although care needs to be applied to make sure that the occurrence of the very short keyword *in* is being handled correctly.

Once the boundary is defined, the parsing continues: the solvent is defined as *THF*, which is a well established abbreviation for *tetrahydrofuran*. The active ingredient requires several more steps: the suffix of *2 M* is taken to be a quantity definition. The capital letter M in this context is shorthand for molar, so the concentration is interpreted as 2 mol/L. Once the quantity information is processed and removed, the remaining text needs to be further truncated: the use of the word *solution* is superfluous, and requires a deletion rule. Once this is done, the remaining text—*trimethyl(trifluoromethyl)silane*—is a legitimate chemical name that can be parsed and converted into a structure.

**Fig. 12:**
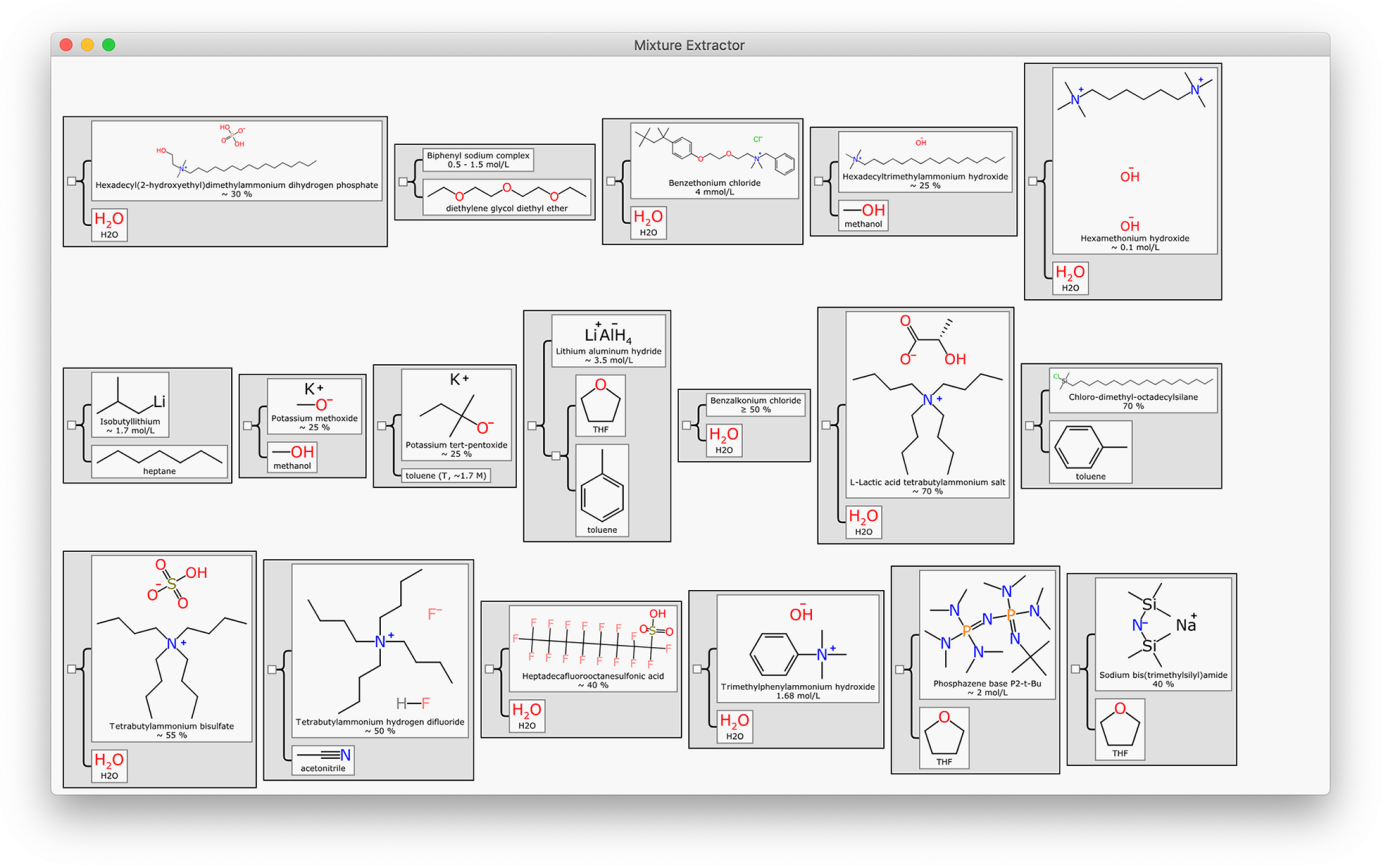
A mosaic of mixture data extracted by applying text extraction from a collection of catalogs

These two case studies are representative of a large number of common text mixture descriptions for laboratory reagents. In the Methods section we describe a brief summary of our ongoing work toward text extraction of mixtures, and the availability of data that we have generated thus far. A collection of several thousand mixture examples is also included within the open source GitHub project, all of which have been generated using our proof of concept text extraction method, some of which are shown in Fig. 12.

The text-to-structure recognition that makes up a key part of the extraction process can be done using one of several available algorithms. For practical purposes it is necessary to combine this functionality with a lookup table, since it is very safe to assume that no algorithm will correctly interpret all of the important structures in any sizeable collection. Furthermore, there are cases where a name is correlated to a sub-mixture (e.g. the ever common *hexanes* and *xylenes*), and these can be handled by providing the lookup table with the ability to insert a mixture branch.

### Mixtures InChI

The Mixfile format that we describe in this article is suitable for use as a reference container, which is appropriate for detailed archiving purposes. It can be easily rendered to create a print quality visual representation, and it can be extended to store any kind of additional metadata beyond the baseline specification. The development of this format and its associated tools have been heavily influenced by our collaboration with IUPAC, and their proposed Mixtures InChI notation, abbreviated as *MInChI*. By design, the Mixfile container representation can be used as the source material to generate a MInChI string, which involves extracting fundamental information about components, and imparting to them the canonical standardisation and layer motif that comes from using InChI as the structure identifier.

**Fig. 13:**
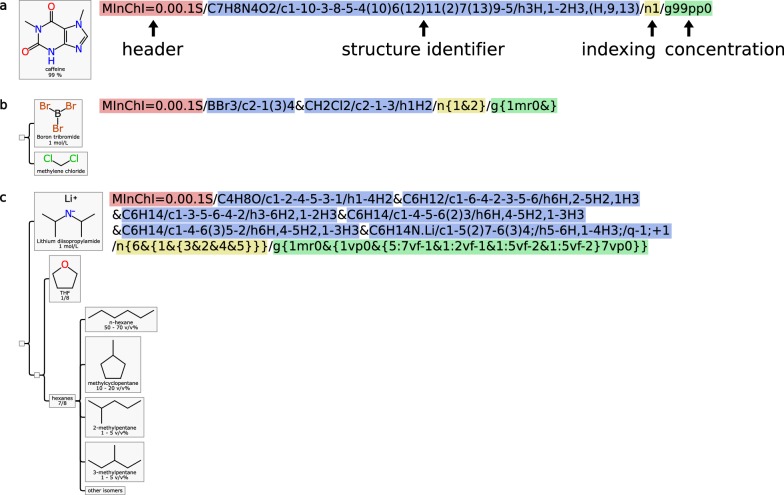
Three increasingly complex examples of mixtures being represented in MInChI notation: **a** caffeine with a purity estimate; **b** boron tribromide dissolved in methylene chloride; **c** lithium diisopropylamide dissolved in a relatively complex mixture of solvents

As can be seen in Fig. 13a, a simple mixture like this example where *caffeine* is listed with a specific purity, the corresponding MInChI string is dominated by the structure identifier from the standard InChI generator. The string is prepended by the signifier that identifies it as conformant to the MInChI specification, and followed by two additional layers: the hierarchy (which is in this case is a singleton), and the concentration which is encoded in a concise mnemonic form.

Example (b) contains two components, which are listed in the structure section. The hierarchy block is indicative of a mixture with a flat hierarchy. In the MInChI string, the component layer is sorted alphabetically by the InChI strings (which coincidently happens to be the same order as was given in the source Mixfile). The concentration block has one section for each component, but the second entry is blank, since the concentration is not indicated (i.e. presumed to make up the remainder of the mixture).

Example (c) is somewhat more exotic, as a mixture with multiple sets of components with 3 levels of hierarchy. Additionally, 3 of the component nodes have no structure specified. In this case the branch ordering differs from that used in the Mixfile. The hierarchy indexing portion of the MInChI string denotes the shape of the tree using curly braces. Three of the nodes have specified concentrations: the lithium diisopropyl amide ingredient has an overall molarity, and the THF/hexanes constituents are expressed as proportions, which apply specifically to the portion of the hierarchy (i.e. the actual definition of *hexanes* in this example is enumerated explicitly by its structures, and their approximate concentrations relative to each other are defined within their own branch).

While both Mixfiles and MInChI’s are used for the same kinds of data, they serve distinct roles within the overall cheminformatics infrastructure. The MInChI notation has some key benefits relative to the originating Mixfile:

- it is concise, limited to a single line made up of ASCII characters, which can be easily manipulated in a spreadsheet or pasted into a single input line on a web form
- it enables easy reference for similarity comparison: two mixtures with the same constituents will be identical up to the *indexing* and *concentration* sections
- testing for the presence of a structure within a mixture is extremely easy (e.g. whether the query InChI identifier is contained within the MInChI string)
- similarly, structures can be separated out and indexed individually by their InChI codes
- relatively sophisticated comparisons of composition and concentration can be made using simple string manipulation, without the need for a dedicated cheminformatics library

These characteristics are all relevant for implementation in a database, where user search queries and indexing operations can be carried out using built in operators or simple scripting languages, which do not always have convenient cheminformatics libraries readily available. Providing the ability to search for a single structure within any mixture becomes very simple (any implementation of string *indexOf* will suffice, as long as the query structure can be converted into an InChI identifier).

**Fig. 14:**
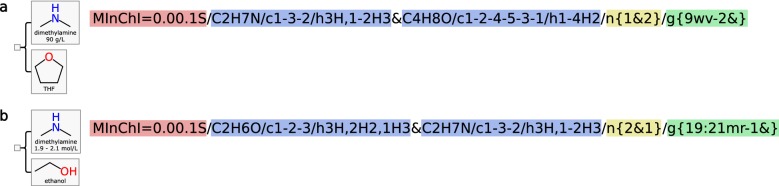
Two very similar mixtures and their corresponding MInChI notation, highlighting the ease with which they can be analyzed with basic string processing

Performing comparisons between mixtures can be achieved with some relatively straightforward logic. Consider a scenario where a database is being searched for mixtures that are similar to the query, shown in Fig. 14(a), and considering (b) as the potential candidate. Both of these mixtures represent *dimethylamine* at an analogous concentration, dissolved in two different solvents. Comparison of the two MInChI strings can quickly establish that each mixture has two components, and they share one in common. The common structure, which is the active ingredient (with an InChI fragment of C2H7 N/c1-3-2/h3H,1-2H3), is given a concentration on both sides: for (a) it is specifically 90 g/L, whereas for (b) it is between 1.9 and 2.1 mol/L. Because the InChI identifier fragment begins with the molecular formula, it is straightforward to calculate the molecular weight (using a very simple lookup table for the elements, and a very short block of code). This can be used to ascertain that 90 g/L is approximately 2 mol/L, and so both of these mixtures have a common ingredient with a common concentration, with a different solvent.

As with the standalone structure identifier (InChI), there are usually compelling reasons to retain the more detailed source information, e.g. consider the MInChI string as a composition notation that is regenerated from a Mixfile, because it is not intended to be the primary record of data. The MInChI generation process abstracts the structure identifier, concentration and proportional relationships of the components as stated in the original description. Both the MInChI string and its constituent InChI identifiers are only reversible in a partial sense: converting forward (i.e. *Mixfile* to *MInChI*, or *Molfile* to *InChI*) reduces the degrees of freedom in order to improve its utility for specific purposes. Any given MInChI or *InChI* string can correspond to numerous different-but-equivalent expressions of a mixture or structure, but reversing the transformation generally does not rederive the original input. In the case of the InChI identifier, this is easy to observe, since InChI does not preserve the coordinates of the input molecules, so the reverse process must recreate them algorithmically. Other modifications, like picking a canonical tautomer, normalising stereocentres and disconnecting bonds to metals further reduce the correlation to the original input structure. In addition, for the Mixfile to MInChI transformation, properties such as structure names, auxiliary identifiers, etc., are not stored in the MInChI notation. It may sometimes be possible to rederive these, but there is no guarantee that they will be the same as the original.

This unidirectional reduction of information is key to the practical value of InChI and all of its derivatives: being able to treat a string as a uniquely and literal definition for a chemical entity makes a great many complex and resource intensive cheminformatics tasks almost trivially simple. The MInChI notation leverages these fundamental InChI properties. The caveat is that an archiving system is advised to also store data in its original form, prior to any original processing, which is a familiar maxim of science (i.e. never throw out the original laboratory notebook).

At the time of writing, the MInChI specification is nearing Phase 1 completion, and is expected to be formally released later in 2019. Updates will be posted on the IUPAC project page [17]. If you are interested to implement MInChI notation in your local systems, please contact the authors.

## Methods

### Format

The GitHub repository at https://github.com/cdd/mixtures provides a working implementation of the Mixfile format, a graphical editor, a MInChI generator, and a collection of sample data, among other things. All of the contents (source code, data, protocols) are made available to anyone under the terms of the GNU Public License (GPL) v3. This article describes a snapshot in time during the development of the project, which continues to progress. The latest content within the repository can be considered current and definitive at any given time.

The Mixfile format that we introduce in this article is deliberately minimalistic and simple. Because it uses JSON [18] as the encoding method, implementing basic read/write/modify operations is simple and convenient in most modern programming languages. JSON is a very popular serialisation technique, and it is not only ubiquitous and easy to manipulate programmatically, but is also relatively concise and human readable [19, 20]. More detailed editing and analysis introduces the dependency on the *Molfile* (aka CTAB) structure representation, and compliance with the specification rules for specifying quantities.

The very basic outline of the Mixfile JSON format (version 1.0) is:
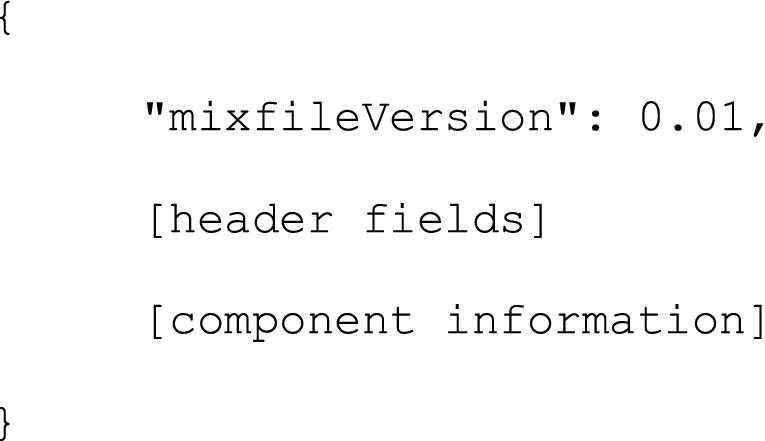


The [header fields] are all optional extensions, and allow additional information about the mixture as a whole. This could include information such as the calculated MInChI string, the original full name of the mixture, inventory tracking information, etc. In a later version some of these may be codified, but for now they are purpose-specific and are not interpreted by the reference editor tool.

The [component information] has the following outline pattern:
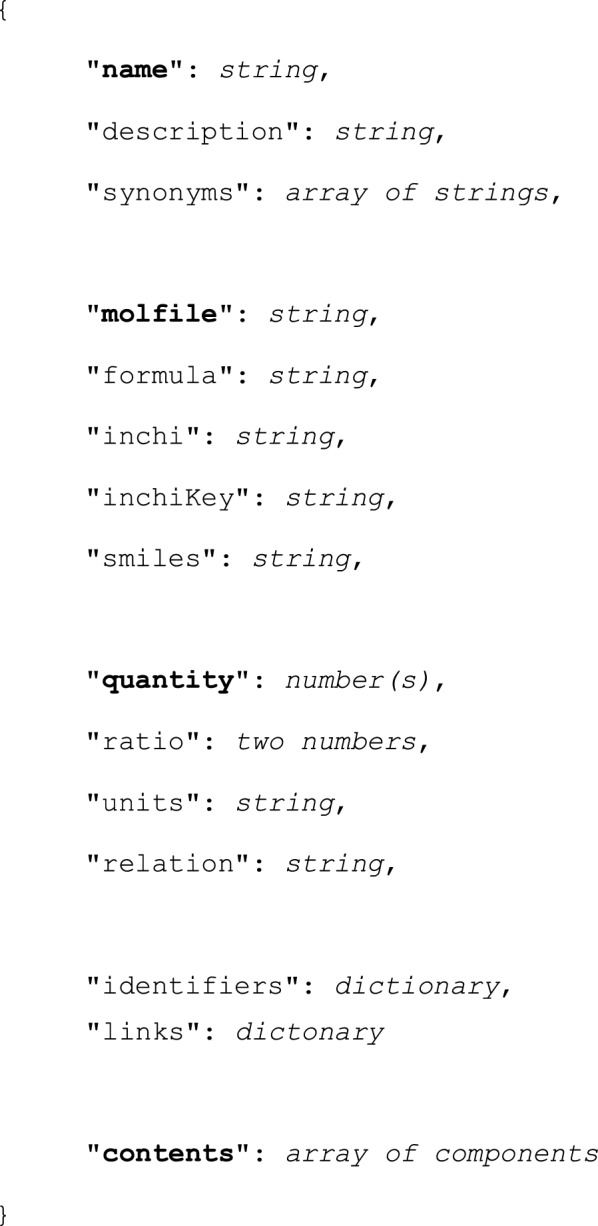


The component definition is divided into 5 categories: *name*, *structure*, *concentration*, *reference* and *sub*-*components*.

The *name* of a component should be provided when possible. For specific structures, this should be a common name or an IUPAC name that matches the structure. In cases where the structure is unknown or not applicable, the name can be any concise moniker that has been used to label the substance. It should be short and fit on a single line. The *description* field can be used to provide more detailed information about the substance, and can be made up of sentences or paragraphs: it should be assumed that it will not be rendered in full when composing a diagram for the mixture, so verbosity is appropriate. The *synonyms* array is an optional list of alternative names. The *name* field itself should contain the preferred choice, but most commonly used chemical substances are referred to in several different ways, and they can be stored here.

The preferred way to define a chemical structure is to populate the *molfile* field, which is a string that conforms to the Molfile CTAB format [21]. In principle all non-query features of the format are allowed, but in practice it is recommended that as small as possible a subset of the V2000 definition is used, in order to ensure compatibility with as many software packages as possible. There are four related fields that can be defined within the component: *formula*, *inchi*, *inchiKey* and *smiles*. These auxiliary fields should be considered as transient properties, to be derived from the *molfile* field: whenever the *molfile* is changed, the dependent values must be recalculated (or if this is not possible, they should be erased). The exception is for scenarios where a mixture was composed from data for which there is no Molfile CTAB representation available, but there is information about the composition of the structure (i.e. formula/InChI/SMILES).

The *concentration* section consists of several interrelated fields: for the most common case, *quantity* and *units* are both provided. The *quantity* field can be given as a single number or as a range (an array of two numbers, e.g. [10, 20] to denote between 10 and 20). The *relation* field can be defined when the quantity number is other than a point value, e.g. inequalities or approximately. Concentrations can also be defined by indicating the *ratio*, which is given as an array of two numbers (numerator and denominator respectively).

Table 1 lists the units that are currently considered valid for Mixfiles, which are stored internally as the common representation. The corresponding URIs from the Units Ontology [22] are given for reference. The translation from Mixfile units to those used by MInChI is shown as the two-character shorthand notation, and the scaling factor. Note that ratio units are handled as a special case, since the Mixfile stores them in [numerator, denominator] form, while in the MInChI notation, only the numerators are listed, and the denominator is implied.

**Table 1:** Standard units for Mixfile and MInChI, shown with corresponding term from the Units Ontology [22]

Common	URI^a^	MInChI	Scale
%	UO_0000187	pp	1
w/v%	UO_0000164	wv	0.01
w/w%	UO_0000163	wf	0.01
v/v%	UO_0000205	vf	0.01
mol/mol%	UO_0000076	mf	0.01
mol/L	UO_0000062	mr	1
mmol/L	UO_0000063	mr	1E−3
μmol/L	UO_0000064	mr	1E−6
nmol/L	UO_0000065	mr	1E−9
pmol/L	UO_0000066	mr	1E−12
g/L	UO_0000175	wv	1E−3
mg/L	UO_0000273	wv	1E−6
μg/L	UO_0000275	wv	1E−9
mol/kg	UO_0000068	mb	1
ratio	UO_0000190	vp	1

^a^URI prefix: http://purl.obolibrary.org/obo/

The *reference* section contains the two optional fields *identifiers* and *links*. Both of these are unconstrained, and so the nature of the content has no specific interpretation policy in the current version of the format. Identifiers are typically database assignments, and can be paired with human-readable keys to signify popular repositories, such as CASRN [23], PubChem [24], ChemSpider [25] and among others. It is also appropriate for calculated identifiers and hash codes (e.g. non-standard InChIs, other kinds of line notations, etc.). The *links* field should be populated with URLs that are relevant to the component.

The final section is the *contents* field: this is an optional array, for which each of the elements follows this component definition. In this way, the Mixfile format can be expressed as a tree, with any desired amount of nesting.

The core functionality of the Mixfile format is delivered using just a handful of fields, i.e. **name**, **molfile**, **quantity**/**units**/**relation**/**ratio** and **contents**. The other fields provide a way to flesh out additional details about the mixture, if it happens to be available. As described above, the current version of the format reserves a handful of additional field names for certain purposes, but they are secondary. As the format evolves, additional fields will be defined, and specific use cases will be added for them. It is valid to add any additional fields for private use, but if in order to ensure that custom fields will never clash with fields that are reserved in future, they can be named by preceding with an underscore.

The aforementioned GitHub repository contains a working implementation of the Mixfile format, which is written in the TypeScript [26] language. TypeScript is a more expressive version of JavaScript that adds compile-type typing, and so datastructures like the Mixfile itself can be expressed as a source-code pattern [27] in much the same way as classes are defined in other object oriented languages. Because TypeScript cross-compiles to JavaScript, the core functionality can be incorporated into a web page. The Mixfile Editor tool is delivered as an Electron [28] app, which means that it runs in the same way as a conventional Windows/Linux/macOS application. The documentation in GitHub repository should be consulted for further information on installing, configuring, deploying and getting involved.

### Text extraction

The text-to-mixture extraction process eluded to in the Results section was carried out with three main functional pieces: a collection of rules using regular expressions, name-to-structure using OPSIN [29], and a lookup table. The parsing rules contain regular expressions for identifying concentrations, splitting blocks of components and removing superfluous content. OPSIN is used for exploratory detection of chemical names, and subsequent conversion into interpretable chemical structures. Having a lookup table serves two purposes: structure names that cannot be identified by OPSIN can be prespecified, and likewise for common names for substances that are themselves mixtures (e.g. solvents like *xylenes* or *hexanes*).
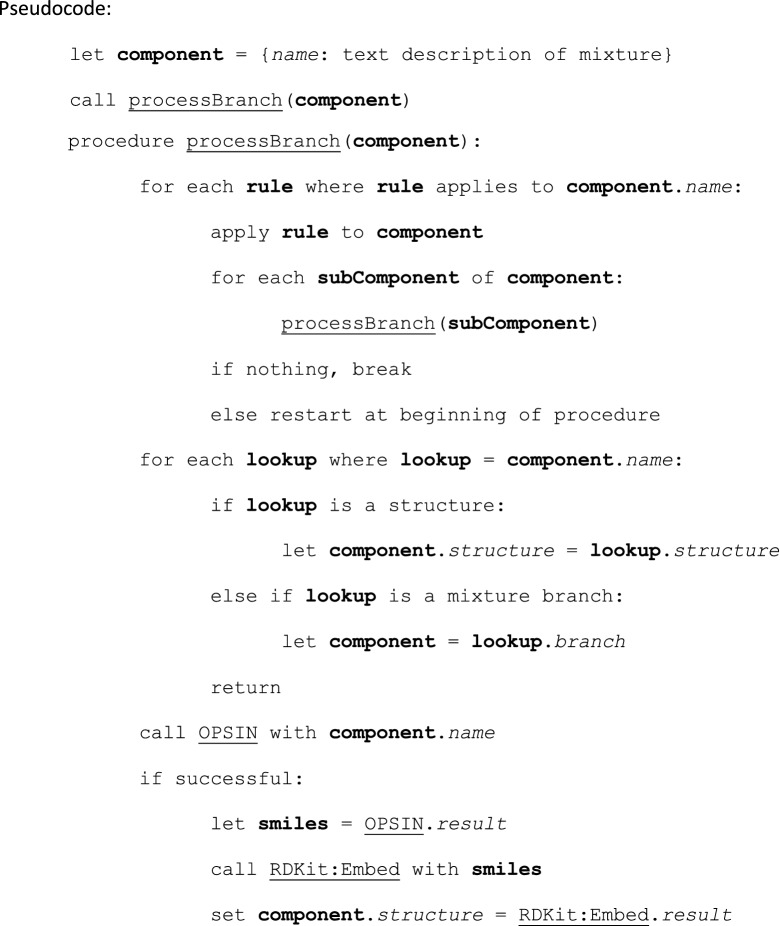


The algorithm is a recursive procedure that begins with a root component, for which the *name* field is set to the full text of the mixture description. In the null case where none of the operations apply, the resulting mixture is a single node with the unmodified text as the name.

Each level of processing is divided into 3 sections, which look for ways to convert the name into something with more metadata. The first step involves iterating through all rules, which are *applied* to the current mixture component whenever a match is found. Each rule is based on a regular expression, and there are several different types:

- Remove: delete superfluous content from the name
- Replace: modify the name by substitution
- Concentration: identify and assign concentration quantity/units/relation
- Branch: split the name into two or more parts, each of which becomes a sub-branch

Note that when the *Branch* rule is triggered, the algorithm becomes recursive, because new sub-components are created, each of which has its own *name* definition, which is processed in the same way.

Once no more rules apply to the name, it is checked against all names in the lookup table. Entries with a given structure are used to copy over the *molfile* field, while entries with a mixture branch definition overwrite the component completely. These often contain their own sub-branches (e.g. the definition for “xylenes” explicitly enumerates out the ortho, meta and para isomers).

If the name is not found in the lookup table, it is submitted to the OPSIN program, which is run externally. When successful, OPSIN returns a SMILES string. In order to convert this into the desired *molfile* format, with a generally reasonable 2D layout, a short Python script is used to invoke the corresponding functionality from the RDKit [30] library.

Source material for text extraction was gathered from several different publicly offered commercial catalogs and combined with internal data from inventory systems operated by collaborators [31]. The rules and lookup table entries were handcrafted, and matches were validated individually. The process was continued until the list of parsed results was deemed to be sufficiently large and representative of common laboratory mixtures.

### MInChI Generation

The algorithm for conversion of Mixfile instances to MInChI strings generates output that is compliant with the current draft specification. The first step is to iterate over all components and ensure that there is an available standard InChI identifier for each instance where a structure is available. This is done by executing the InChI command line tool.

Assembling the MInChI string [32] involves building the 3 primary layers that make up the result:

MInChI = 0.00.1S/**components**/n**indexing**/g**concentration**

The *components* are collected by iterating over each component and adding its InChI to a set, if it has one. The set is sorted, any duplicates are removed, and each of the InChI strings (sans prefix) are concatenated together with the ‘&’ character as the separator. At this stage of the assembly, a mixture of *formaldehyde* and *water* would be:

MInChI = 0.00.1S/**CH2O/c1-2/h1H2**&**H2O/h1H2**

Once the composition and order of the eligible components is established, the algorithm then iterates over the hierarchy of the mixture. Both the *indexing* and *concentration* layers are built at the same time, in the same order: both of these layers use curly braces to denote traversal down a branch, and use the ‘&’ character to separate two adjacent component nodes.

At each step of the traversal through the tree follows the logic:

1. if there are any child nodes:
  call the traversal function for each of them
  for *indexing* and *concentration* layers, append the results, wrapped in curly braces and separated by ‘&’
2. if the current node has an InChI identifier, append its index to the *indexing* layer
3. if the current node has concentration information, append it to the *concentration* layer

The indexing layer for the *formaldehyde* and *water* mixture would be/n{1&2}, where the two numbers refer to the indexes of the InChI identifiers, formaldehyde and water respectively.

Formatting concentration information involves examining the various different permutations for how the quantity/units/ratio/relation fields are used in the Mixfile. The Mixfile format defines valid units that can be mapped effectively to those that are permitted by MInChI, some of which require scaling (e.g. mol/L, mmol/L and μmol/L are all equivalent to MInChI’s mr for molarity once they have been scaled appropriately). Ranges are expressed by separating the two values with the ‘:’ character. Numeric values are converted into scientific notation.

## Conclusions

We have demonstrated the basic tools necessary for capturing mixture data in a way that is analogous to the practice of describing individual compounds as interpretable and renderable Molfiles. As with any new protocol, our proposed Mixfile format will take time to become accepted by the community and achieve significant adoption. In part to grease the wheels, we have made a fully functional editor tool available as an open source, and have extracted a collection of several thousand examples of marked up mixture definitions that are freely available for use as reference material.

Furthermore we have positioned the Mixfile format, and the tools that we have created for using it, as a viable entrypoint to the upcoming IUPAC Mixtures InChI notation. The *Mixfile*:*MInChI* pairing has been intentionally tailored so that the aims of storing and linking original data with all of its context (cf. electronic lab notebooks) and bulk informatics data warehousing can be achieved by using these two complementary descriptions.

The work that we describe in this article is in some ways a minimum viable product: our emphasis on the core features of a mixture (structure, name, concentration and hierarchy) is intentional. With these fundamentals operating for a variety of use cases, other kinds of metadata can be formally codified into the format as it evolves.

While we have considered a variety of use cases for our initial work, the universe of chemical mixtures is truly vast, and it is hard to know for sure where the utility of the Mixfile format and MInChI notation will ultimately taper off. In terms of industry applicability, most of our development and testing has been to ensure that mixture descriptions are useful in a research laboratory environment, with inventory management and catalog listing being two use cases that are self-evident: both of these scenarios are suffering from the fact that existing single-molecule cheminformatics tools and auxiliary text can satisfy a fair portion of the demands of an information system, but leave many important use cases unsatisfied.

As the domain of interest expands beyond the chemistry laboratory, there are innumerable applications of mixtures whereby the practitioners are not necessarily in the habit of thinking about the chemical definitions of the materials they are working with. Drug formulations are an important example: while this is still within an R&D context, it is not necessarily common practice to rigorously define the chemical composition, and having an easy means to describe this content and carry it forward is highly valuable. Consumer products (which includes formulated drugs once they have passed the requisite tests) are by definition available to a broad range of customers, some of whom have the desire or need to find out what they are made of. Requirements for reporting and internal process control can definitely benefit from having standardised and widely supported data description protocols. The current practice of listing ingredients on the label leaves much room for improvement, and would be greatly improved by having a way to look up a more thorough, precise and machine readable definition.

The design of both the Mixfile and MInChI formats is intended first and foremost to capture the composition of a mixture from a *recipe* perspective, i.e. to record the components and their relative proportions as they went into the mixture. This is the reason why both formats support a hierarchical tree structure, as an alternative to just enumerating the constituents in a flat list: the hierarchical form naturally captures elements of the mixing process, e.g. when a chemist dissolves a substrate in a solvent from a bottle labelled “hexanes”, it is not the same workflow as measuring out a series of aliquots of C_6_-hydrocarbons. While the Mixfile does not currently define any way to record any additional experimental distinctions, just the fact that *hexanes* can be defined as a specific thing, which is itself defined as a mixture of other things, is important information that is worth recording. Another useful nuance is that sometimes a meta-component (like *hexanes*) has an uncertain composition, which can be expressed by ranges or approximate concentrations. Confining this uncertainty to one section of the mixture definition is useful, because other parts of the mixture (e.g. the molarity of the active ingredient) may have been measured precisely. Partitioning the mixture into a hierarchy allows uncertainties to be localised relative to the associated components within the appropriate branch.

Just because the recipe workflow drove the original use cases does not mean that is all they are useful for: we envisage that Mixfile and MInChI will be equally useful on the other side of the mixing process—for analytical standards. When an instrument is used to measure the concentrations of some number of species, the results can be effectively captured with either or both of these formats. This use case would not necessarily need to use the hierarchical composition capability (or if it did, it would not be used to denote the way in which it was originally mixed). The ability to specify explicit chemical compounds, their concentrations with optional uncertainty, and the flexibility to omit unknown components are all functional features that are relevant to the recording of analysis results.

For encouraging adoption of the Mixfile format (and hence implicitly adding the value of MInChI), there are three main places where the generation of Mixfile-formatted data can be interjected:

- creation of new data during electronic lab notebook (ELN) authoring, e.g. using the open source editor
- programmatic creation when the preexisting information is known
- text extraction of existing collections of mixtures

Freshly created data can be anticipated at many different points in the workflow of a scientist working at the bench: whenever the purity of a material is estimated, or a solution is prepared, this is an opportunity for the ELN software to prompt the scientist to indicate the material as being a mixture, with the requisite information known. Ideally the software will need to be as innocuous as possible (which is no different to any other kind of data capture UI/UX design). Achieving this will require the enhancement of existing ELN tools, or in some cases merely the formalisation of tools that are already capable of capturing this information (i.e. storing as Mixfiles rather than an ad hoc format). Augmentation of the CDD ELN product is currently being explored [33].

Programmatically created data is similarly varied: there are numerous cases where software is being used under conditions where constituent materials are known. For example, if a formulations chemist is composing a model for optimising the components of a tablet, the materials and concentrations may be listed as columns in a spreadsheet. These can be converted into a Mixfile using a simple script, as long as it is run before the institutional knowledge evaporates (i.e. the meaning of each of the columns and their contents is still known by the author of the script).

A common problem with informatics disciplines, to which cheminformatics is no exception, is that it is often difficult to demonstrate the value of having data in a machine readable format until a substantial amount of data has already been curated, presenting a chicken vs. egg paradox. As with previous projects [34, 35] we have endeavoured to frontload this project by using text extraction. The relatively simplistic method we describe briefly in this work has been effective enough to create a baseline collection of mixture descriptions, which could be used as the initial population for a mixtures database. Such a service would be a valuable supplement to the dozens of major public databases that index primarily on individual chemical structures.

In principle, any organisation with mixture data could implement software tools such that all *new* content generated would conform to the machine readability standards of a Mixfile, meaning that their legacy data is a finite resource which only needs to be curated once. The practical reality is that starting with a largely automated conversion process is the most effective way to get started. For this reason we are currently exploring the possibility of designing a much more sophisticated extraction tool, which will create its own machine learning model for partitioning and annotating the name/structure/concentration sections of a text mixture description, as opposed to using a predefined set of rules. This approach would use an existing training dataset (text + Mixfile), which would allow us to design an interface whereby the operator can confirm or correct the predicted outcomes, until the model is sufficiently accurate for purpose.

The initial definition of the Mixfile format mandates that the Molfile CTAB V2000 format is the only available method for structure representation (with the option of also using *identifiers* like InChI or SMILES). The Molfile format is the only realistic choice because it is the dominant industry standard, but it does come with a large amount of baggage. The latest version of the specification, if implemented correctly, can be used to describe a reasonably large proportion of all of the molecular species that are likely to be encountered as mixture components. Unfortunately the subset of these features that is reliably implemented by most major software packages is small, and typically limits structures to monomeric organic compounds and a small fraction of inorganic molecules. There is some temptation to define the compatibility list to match structures for which the InChI algorithm can generate a meaningful identifier, but this is not a rigorous definition. The process of clarifying which Molfile CTAB features should be part of the pragmatic industry standard is ongoing within the cheminformatics community, and the intention is for the Mixfile specification to evolve with it.

Beyond troublesome representational details for well defined isomers of monomeric compounds, there are whole new categories of issues that arise with isomer combinations and polymers. Isomers are usually not an issue for a mixture format, since the basic purpose is to provide a well defined way to enumerate individual compounds. For practical purposes, any mixture of stereoisomers that can be accurately denoted with the *squiggly bond* convention to indicate R/S or E/Z mixtures may be drawn as a single structure, rather than as an ensemble. For diabolical cases, such as a molecule with dozens of stereocentres and very specific rules as to which are valid, there is currently no good solution except to enumerate them all, even if the list is unwieldy. Other phenomena such as numerous interconnected tautomerisation options also must be treated by enumeration, if it is necessary to ensure that the right species are represented. Some of these enumeration preferences clash with the downstream MInChI conversion, e.g. normalisation of tautomers, which is a subject that is being hotly debated within the community.

And of course enumeration of polymers as a collection of all extant molecular species is not viable: there is no alternative but to define a formula for how to join one or more fragments together to form a distribution of very large molecules. The Mixfile format, and the tools that have been built for it so far, defines no such capabilities. This is however an important item to consider for the medium term roadmap, especially for polymers, because these frequently appear within mixtures (either as primary ingredients or adjuncts). At the present time it is necessary to define them by names or database identifiers, which are not inherently machine readable.

Additionally, there are also many kinds of materials that are not well described by 2D sketches which may benefit from the ability to include in mixtures. One example is ceramics, which could be defined by non-integral element counts (e.g. La_1.85_Ba_0.15_CuO_4_). Materials with non-obvious molecular structures are often defined by a diverse collection of properties, none of which fits into the relatively convenient mould of a Molfile CTAB, and so expanding to include such entities will take some effort.

Generally speaking, the overall value proposition for upgrading chemical mixtures to a descriptive machine readable format is large. We are proposing to do this as a long term series of milestones, beginning with the data that is most similar to what is already within the realm of cheminformatics.

## Data Availability

All materials mentioned in the manuscript as being openly available can be found in GitHub, as mentioned in the text.

## Abbreviations

InChI: international chemical identifier
MInChI: mixtures InChI extension
